# Antiretroviral treatment and association with prematurity in perinatal HIV-exposed children

**DOI:** 10.1186/1471-2334-14-S7-O9

**Published:** 2014-10-15

**Authors:** Ana Maria Tudor, Mariana Mărdărescu, Ioana Alina Anca, Cristina Petre, Ruxandra Neagu Drăgchicenoiu, Rodica Ungurianu, Dan Oțelea, Simona Paraschiv

**Affiliations:** 1 Carol Davila University of Medicine and Pharmacy, Bucharest, Romania; 2 National Institute for Infectious Diseases "Prof. Dr. Matei Balş", Bucharest, Romania; 3 The Institute for Mother and Child Protection “Alfred Rusescu”, Bucharest, Romania

## Background

In the last years there is a great concern regarding the effect of HIV and antiretroviral drugs in children born by treated HIV-infected mothers.

We started a prospective cohort study regarding HIV and antiretroviral exposure in children followed in the Pediatric HIV Department from the National Institute for Infectious Diseases "Prof. Dr. Matei Balş", Bucharest.

## Methods

We analyzed the data recorded for children perinatally-exposed to HIV followed up in our Department from January 1^st^ 2006 to December 31^st^ 2012. The patients were followed up for 18 month after birth to establish the HIV status; gestational age, birth defects and mother treatment were noted.

## Results

From 206 children with complete 18 months follow up, 21% (43 cases) were diagnosed with HIV infection and more than 33% had at least one congenital condition.

We found birth defects in various organs in studied children: heart (130 cases), musculoskeletal system (47 cases), kidney (20 cases), nervous system (20 cases), digestive tract (10 cases) and metabolic and genetic disorders (2 cases each). 26 from the 163 HIV-exposed children and 6 from the 43 HIV-infected cases were born before 37 weeks of gestation, 4 HIV-exposed and 4 HIV-infected children were small for gestational age. We found low birth weight (<2500 g) in 18 HIV-exposed children and 3 HIV infected children and extremely low birth weight (1000) in one HIV-exposed child.

We found congenital malformation in 11 preterm HIV-exposed children and 2 preterm HIV-infected children, but also in 38 HIV-exposed children and 19 HIV-infected babies with normal gestation period.

The difference between the rate of congenital malformation and prematurity was not statistically significant (p=0.08) in any of studied groups and HIV diagnosis was not associated with a higher risk of preterm birth (p=0.93). Mother being part of the Romanian cohort was not statistically associated with higher risk of prematurity. We found a significant association between antiretroviral treatment during pregnancy and prematurity (p=0.003). The most used drugs to treat mothers were boosted protease inhibitors (99% cases).

## Conclusion

In the studied patients we found high risk of prematurity in babies exposed in utero to antiretrovirals, but no association between prematurity and HIV infection in children or mother being part of Romanian cohort.

